# Emerging concepts in the molecular cell biology and functions of mammalian erythrocytes

**DOI:** 10.1016/j.jbc.2025.108331

**Published:** 2025-02-19

**Authors:** Sangeetha Devi Kumar, Japita Ghosh, Swati Ghosh, Sandeep M. Eswarappa

**Affiliations:** Department of Biochemistry, Indian Institute of Science, Karnataka, Bengaluru, India

**Keywords:** erythrocytes, RNA, innate immunity, blood, blood vessels

## Abstract

Erythrocytes, or red blood cells, are essential components of vertebrate blood, comprising approximately 45% of human blood volume. Their distinctive features, including small size, biconcave shape, extended lifespan (∼115 days), and lack of a nucleus or other membrane-bound organelles, make them unique among mammalian cell types. Traditionally regarded as passive carriers of oxygen and carbon dioxide, erythrocytes were long thought to function merely as hemoglobin-filled sacs, incapable of gene expression or roles beyond gas transport. However, advancements in molecular biology have revealed a more complex picture. Recent studies have identified various RNA types within erythrocytes, demonstrated globin mRNA translation, and uncovered miRNA-mediated defenses against *Plasmodium* infection. Beyond gas exchange, erythrocytes play critical roles in regulating regional blood flow *via* nitric oxide, contribute to innate immunity through toll-like receptors, transport amino acids between tissues, and maintain water homeostasis. Furthermore, emerging technologies have repurposed erythrocytes as drug-delivery vehicles, opening new avenues for therapeutic applications. This review highlights these recent discoveries and explores the expanding functional landscape of erythrocytes, shedding light on their multifaceted roles in physiology and medicine.

## Erythrocytes

Some of the largest and most complex organisms that ever lived on the earth are vertebrates. Large size and high complexity were possible primarily because of their efficient respiratory and cardiovascular systems, which provide sufficient oxygen and nutrients to all tissues. Erythrocytes (also known as red blood cells) have played a crucial role in this aspect by enhancing the oxygen-carrying capacity. They transport oxygen from the lungs to tissues using hemoglobin (Hb) as the oxygen-binding molecule, which is made up of two alpha globin chains, two beta globin chains, and four heme groups. All known vertebrates have erythrocytes except icefishes that belong to the family Channichthyidae (1). In humans, erythrocytes are the most abundant cells (2, 3). They occupy about 36 to 54% of blood volume (4). Mammalian erythrocytes are unique cells in many aspects. They are one of the smallest cells in humans with a biconcave shape. Unlike erythrocytes of nonmammalian vertebrates, erythrocytes of mammals lack all membrane-bound organelles including the nucleus. These properties help them squeeze through the capillaries, which are narrowest in mammals (5).

Erythrocytes are generated from hematopoietic stem cells by a process called erythropoiesis. In humans, this process takes place in the yolk sac during embryonic stage, in the liver during fetal stage, and in the bone marrow after birth. Hematopoietic stem cells differentiate into a series of erythroblasts, which are erythroid progenitor cells. The differentiation process is characterized by the gradual accumulation of Hb, reduction in size, and condensation of the nucleus. Enucleation of orthochromatic erythroblasts results in reticulocytes, which finally lose the rest of the internal organelles, and remodel their membrane to become biconcave erythrocytes in the circulation (see Ref. (6) for a review). About 2 to 3 million erythrocytes are produced in a human body every second (7). Aberrations in erythrocyte number, size, shape, and content are characteristic features of several pathological conditions such as anemia where the erythrocyte number or Hb amount is inadequate, polycythemia (also known as erythrocytosis), where erythrocytes are in excess, hereditary spherocytosis where erythrocytes are spherical, and sickle cell disease, where erythrocytes attain sickle shape (8, 9, 10, 11).

The process of erythropoiesis is tightly regulated. Erythropoietin is the key regulatory factor of erythropoiesis. It is a hormone primarily secreted by specialized stromal cells in kidneys called Norn cells (12). Hypoxia stabilizes the transcription factor hypoxia-inducible factor-2α in these cells to induce the expression of erythropoietin, which in turn enhances erythropoiesis by accelerating the differentiation of erythroid precursor cells. Thus, erythropoietin ensures the availability of sufficient number of erythrocytes to meet the oxygen demand of our body. Once formed, human erythrocytes stay for about 115 days in circulation (13). Aged and abnormal erythrocytes are marked by increased phosphatidyl serine and reduced CD47 on their surface, which triggers their removal by macrophages in the spleen and liver by a process called erythrophagocytosis (14, 15, 16).

One of the most intriguing facts about erythrocytes is that only mammalian erythrocytes lack the nucleus; all other vertebrate erythrocytes have the nucleus. The evolutionary reason behind the loss of the nucleus from mammalian erythrocytes is not clear. However, the absence of the nucleus seems to have helped mammalian erythrocytes to reduce their size. The small size offers two advantages: it enables erythrocytes to squeeze through the very narrow capillaries of mammals, and it increases the surface area to volume ratio, which in turn allows rapid oxygenation of tissues (17, 18, 19, 20).

There are multiple lines of evidence to suggest that the erythrocyte size is driven by the oxygen demand of organisms. In fact, there is an inverse correlation between these two parameters. A study conducted on Lepidosauria (a superorder in reptiles) observed that species that live in high altitudes and that have warmer body temperatures have smaller erythrocytes (21). Similarly, birds that migrate long distances and that show fierce male-to-male competition have smaller erythrocytes compared with those that do not show these behaviors. On the other hand, birds that dive into the water have larger erythrocytes, which help them to store oxygen and release it slowly (22). Fishes that have a low metabolism rate and, therefore, low oxygen demand, have larger erythrocytes (23). These observations strongly suggest that the behavior and metabolism are influenced by the size of erythrocytes and *vice versa*.

## Molecular biology of erythrocytes: emerging concepts

**Figure 1:**
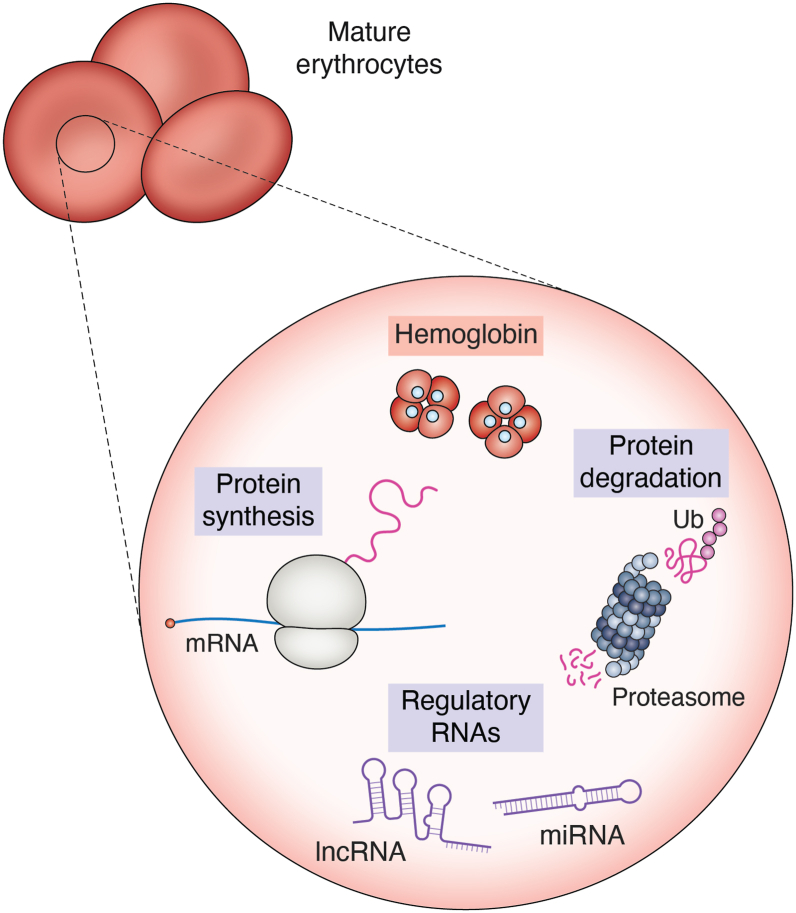
**Erythrocytes exhibit protein synthesis and degradation**. Human erythrocytes are anucleated and, hence, were believed to be devoid of gene expression mechanisms. Recent developments provide strong evidence for the presence of different types of RNAs, functional ribosomes, and proteasomes in these cells.

As mammalian erythrocytes lack the nucleus, for many years, researchers assumed these cells were devoid of nucleic acids as well as gene expression machinery (24, 25, 26). All the required proteins, including the abundant globins, were believed to have come from their precursors, including reticulocytes. However, it was not known how erythrocytes survive and function for 115 days without a fresh supply of proteins. Their close association with oxygen creates constant oxidative stress, which can damage proteins leading to their degradation. Notably, erythrocytes have functional proteasomal degradation machinery (27, 28). As proteins such as globins are erythrocyte specific, they cannot be obtained from neighboring cells such as leukocytes and endothelial cells. Hence, logically, the only way to replenish their proteins is to make them *de novo* inside erythrocytes. However, this is not possible if they do not have RNA. Several studies reported in the past decade provide compelling evidence for the presence of protein synthesis and degradation in erythrocytes (Fig. 1).

### RNA in erythrocytes

The presence of RNA in erythrocytes was first reported by O'Brien *et al.* in 1990 (29). They observed the presence of Ro–ribonucleoprotein complex containing Y1 and Y4 RNAs, which are small noncoding RNAs important for the initiation of chromosomal DNA replication in mammalian cells (30). However, the functional significance of their presence in erythrocytes, which lack DNA, is not known. The advent of sensitive RNA detection techniques such as microarray and next-generation sequencing has led to the identification of several RNAs in erythrocytes in recent years. Mature erythrocytes contain about 2 to 3 × 10^−4^ pg of RNA per cell. Though this is similar to that in platelets, it is much less compared with nucleated cells, which have about 5 to 9 pg per cell (31). The RNA expression profile of mature erythrocytes is different from that of leukocytes and even from reticulocytes, their immediate precursors.

The detection of mRNAs in erythrocytes was first reported by Doss *et al.* in 2015 (32). They isolated erythrocytes and detected several mRNAs, miRNAs, and even long noncoding RNAs using next-generation sequencing (Fig. 1). Among them, mRNAs encoding multiple translation factors and tRNA synthetases and several ribosomal RNAs were found. Gene Ontology analyses revealed the enrichment of RNAs functionally related to protein synthesis. These observations gave the first indication toward the presence of protein synthesis machinery in erythrocytes. mRNAs that encode ubiquitin, ferritin light chain, synuclein alpha, surface protein band 4.1, glycophorin C, ornithine decarboxylase antizyme 1, 5-aminolevulinate synthase 2, and adiponectin receptor 1 were present at a level comparable to their levels in peripheral blood mononuclear cells (32).

### Translation

Proteins belonging to translation machinery, including ribosomes, were detected in pure erythrocyte populations in multiple mass spectrometry–based proteome studies (27, 33, 34, 35). Some of the ribosome proteins even showed high erythrocyte likelihood scores (33). However, these observations were thought to be due to imperfect erythrocyte maturation process, or due to possible moonlighting functions, or due to contaminating cells. Furthermore, transport of all 20 protein-coding amino acids, which constitute the essential raw material for translation, into mammalian erythrocytes has been reported (36, 37, 38, 39, 40, 41, 42, 43, 44, 45, 46, 47, 48, 49, 50).

Detection of translation machinery by mass spectrometry in the studies mentioned previously, and mRNAs and rRNAs by transcriptome analyses by Doss *et al.* (32), provided a strong indication toward translation in erythrocytes. Our group experimentally investigated this possibility in human erythrocytes (51). We were able to isolate and visualize 80S ribosomes from erythrocytes isolated from human blood after excluding reticulocytes and other blood cells. Erythrocytes were able to incorporate [^35^S] methionine in their proteome, especially in globins, demonstrating translation (Fig. 1). These observations were supported by ribopuromycylation assay, which measures the incorporation of puromycin in proteins during translation. Interestingly, globin mRNAs (*HBA* and *HBB*) were the most abundant mRNAs in polysomes suggesting their selective translation. Inhibition of translation by cycloheximide or harringtonine reduced the levels of globin proteins in erythrocytes showing the importance of translation in erythrocytes to maintain normal levels of globin proteins in them. Though these experiments demonstrate translation and its importance in human erythrocytes, the level of translation was low, about 10% of that found in reticulocytes (51). However, this level is much higher than the levels expected if this was due to contaminating blood cells, showing that the signal of translation observed in the study was coming from erythrocytes. Further, the contribution of erythrocyte translation is likely to be physiologically significant considering the long life (115 days) and abundance (4–five million per microliter of blood) of erythrocytes.

### Protein degradation

In the ∼115 days-long life of erythrocytes, proteins are likely to get damaged, which requires their safe disposal. Therefore, it is imperative for erythrocytes to have protein degradation machinery. Unlike translation, protein degradation in erythrocytes is well studied. Degradation of oxidation-damaged proteins, including Hb in erythrocytes, was reported in the 1980s (52, 53). Multiple studies have detected proteasome machinery in human erythrocytes by mass spectrometry (27, 34, 54). Mass spectrometry analysis of the lysates from erythrocytes performed by our group detected 32 proteins belonging to proteasomal complex and 21 proteins related to ubiquitin dynamics, including its ligases and hydrolases (51). Cryo-electron microscopy analyses of proteasome particles isolated from erythrocytes showed intact core proteasomes. About 94% of them were 20S, and the remaining were 26S proteasome particles (33). In another study, functional 20S proteasomes were isolated from erythrocytes and visualized using the transmission electron microscope. Inhibition of their function using lactacystin altered the protein content of erythrocytes (28). All these studies show that erythrocytes possess a functional proteasome-mediated protein degradation system (Fig. 1).

### miRNAs in erythrocytes

miRNAs are small RNAs that target specific mRNAs and inhibit their translation and/or induce their degradation. Thus, they are key players in the regulation of gene expression at the post-transcriptional level. Several miRNAs have been detected in human erythrocytes (55, 56). However, it is not known whether they target any endogenous mRNAs inside the erythrocytes.

#### Role in sickle cell disease and malaria

Notably, the erythrocyte miRNA expression profile of healthy individuals is different from that of sickle cell disease patients, whose erythrocytes acquire sickle shape under low oxygen tension because of a mutation in β-globin (glutamine at sixth position to valine) (31, 57). This change in erythrocyte shape slows down, or even blocks, the movement of blood, leading to the pathological features of sickle cell disease. This change in the miRNA profile contributes to the resistance of sickle erythrocytes to malaria parasites (58). Two miRNAs, miR-451 and miR-let-7i, which are enriched in sickle erythrocytes compared with healthy erythrocytes, translocate into *Plasmodium* parasites and inhibit their growth. Blocking the action of these miRNAs using antisense oligonucleotides (ASOs) increases the parasite growth. Remarkably, these miRNAs are covalently incorporated into parasite mRNAs forming fusion products of miRNA and parasite mRNAs (chimeric mRNAs). While miR-451 is fused with *PKA-R* and *PEAMT* mRNAs of *Plasmodium*, the target of miR-let-7i for fusion is *REX1* mRNA in *Plasmodium* (58, 59). These chimeric mRNAs are not capped at the 5′ end, unlike the unfused mRNAs. Because of the absence of capping, the fused mRNAs are not translated. *PKA-R* encodes protein kinase A, essential for the invasion of *Plasmodium falciparum* into human erythrocytes (60). *PEAMT* encodes a phosphoethanolamine–methyltransferase, whose disruption inhibits the survival and growth of *Plasmodium* (61). *REX1* encodes ring-exported protein-1, which is critical for the trafficking of *Plasmodium* virulence proteins to the erythrocyte surface (62). Thus, erythrocyte miRNAs translocate into the *Plasmodium* parasite and bring down the expression of key virulence factors. Hence, erythrocyte miRNAs play critical defensive roles against pathogens. About 100 human miRNAs have been found in *Plasmodium* parasites during the intraerythrocytic developmental cycle (58). The functions of many of them are yet to be determined.

## Functions of erythrocytes beyond oxygen transport

**Figure 2:**
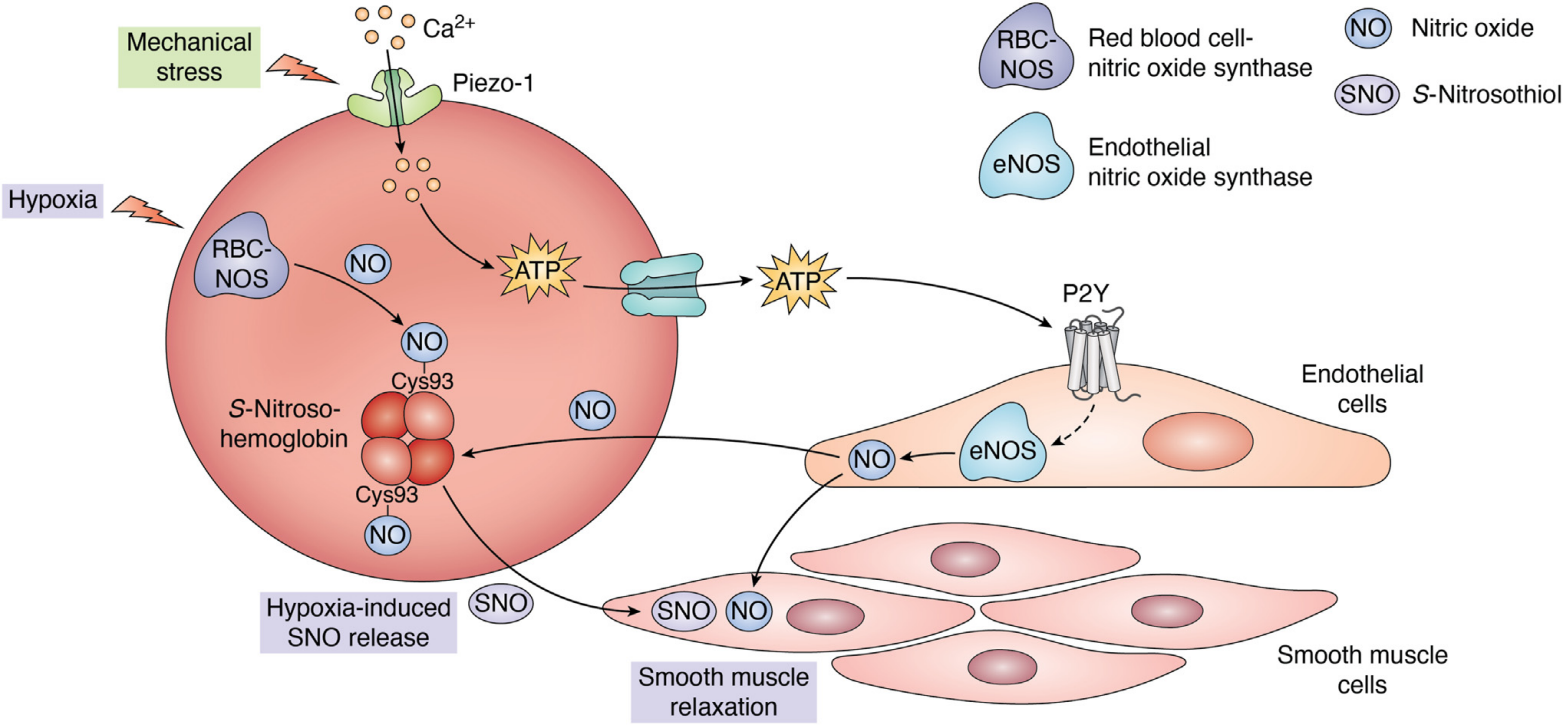
**Erythrocytes regulate local microvascular blood flow**. Erythrocytes possess a functional nitric oxide synthase (RBC-NOS), which produces nitric oxide (NO) in response to hypoxic conditions. The NO interacts with the highly conserved residue βCys93 in the β chain of hemoglobin (Hb), forming S-nitrosohemoglobin (SNO-Hb). NO produced in the endothelial cells can also enter erythrocytes and contribute to the formation of SNO-Hb. Hypoxia induces the release of SNO (S-nitrosothiol), which triggers local vasodilation. This phenomenon provides a mechanism for regulating oxygen delivery depending on the oxygen demand of the tissue. Erythrocytes also regulate the endothelial NOS (eNOS)–mediated blood flow. Shear-induced mechanical stress induces calcium influx *via* Piezo-1 channels. This results in ATP release and induction of eNOS *via* P2Y signaling in endothelial cells. This leads to more NO release, which causes relaxation of vascular smooth muscles and further vasodilation. RBC, red blood cell.

**Figure 3:**
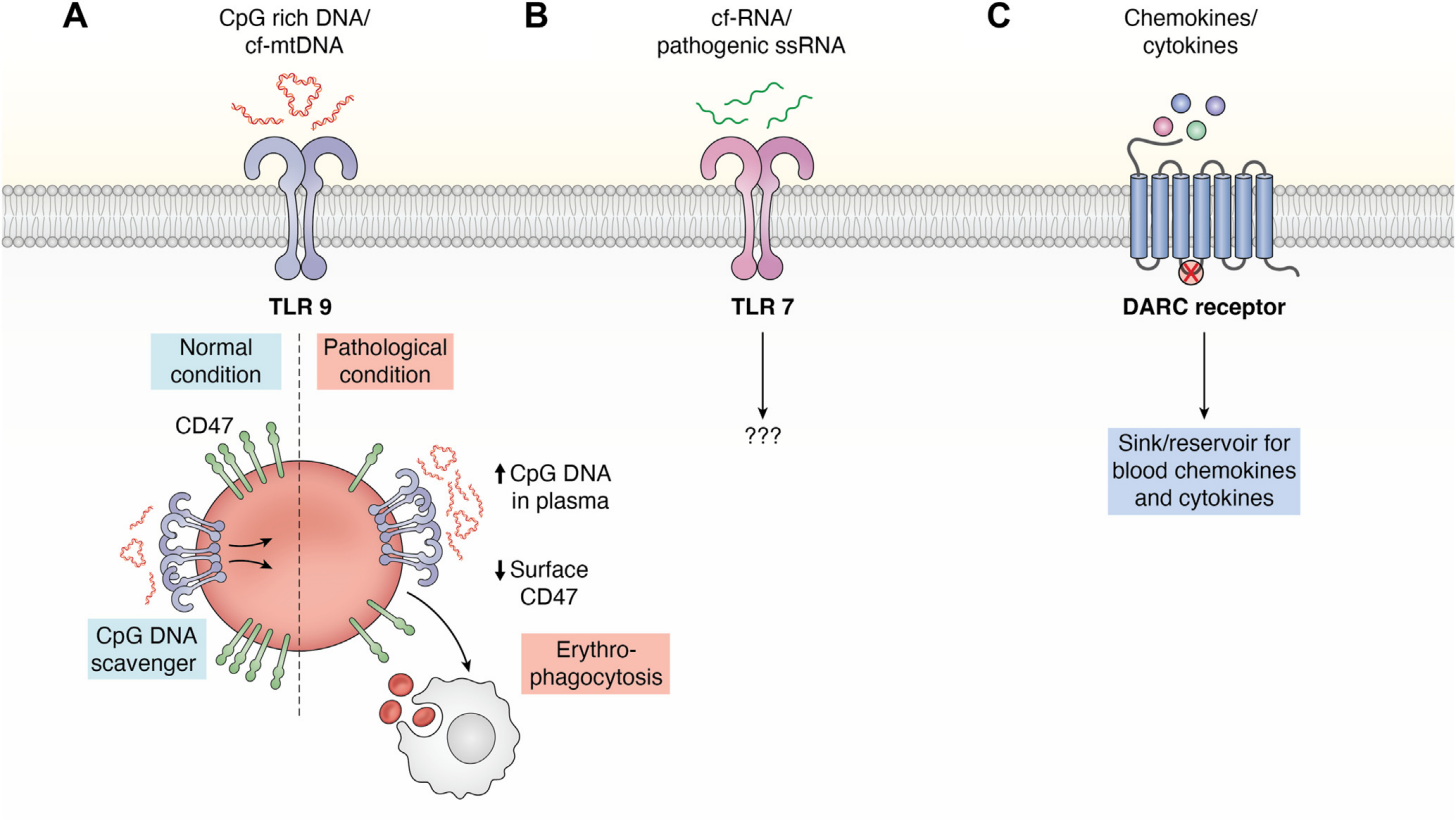
**Erythrocytes regulate innate immune response**. *A*, TLR9 is expressed on the surface of erythrocytes, and it can bind to proinflammatory cell-free mitochondrial DNA (cf-mtDNA) and CpG DNA derived from pathogens. Under normal conditions, TLR9 serves as a scavenger of CpG DNA. However, in pathological conditions such as infections and sepsis, more cf-mtDNA and CpG DNA are found in the circulation, which bind to TLR9 on erythrocytes. This causes enhanced erythrophagocytosis because of loss of surface CD47. *B*, TLR7 is also expressed on the surface of human erythrocytes, and it can bind exogenous synthetic single-stranded RNAs and viral RNAs. However, its significance is not known. *C*, DARC (Duffy antigen receptor for chemokines) is expressed on erythrocyte plasma membrane. It can bind to the free chemokines and cytokines. Due to the absence of a consensus DRYLAIV motif in the second intracellular loop (cross in the figure), it cannot trigger any downstream signaling. Thus, it serves as a sink or reservoir for blood chemokines and cytokines. TLR9, toll-like receptor 9.

**Figure 4:**
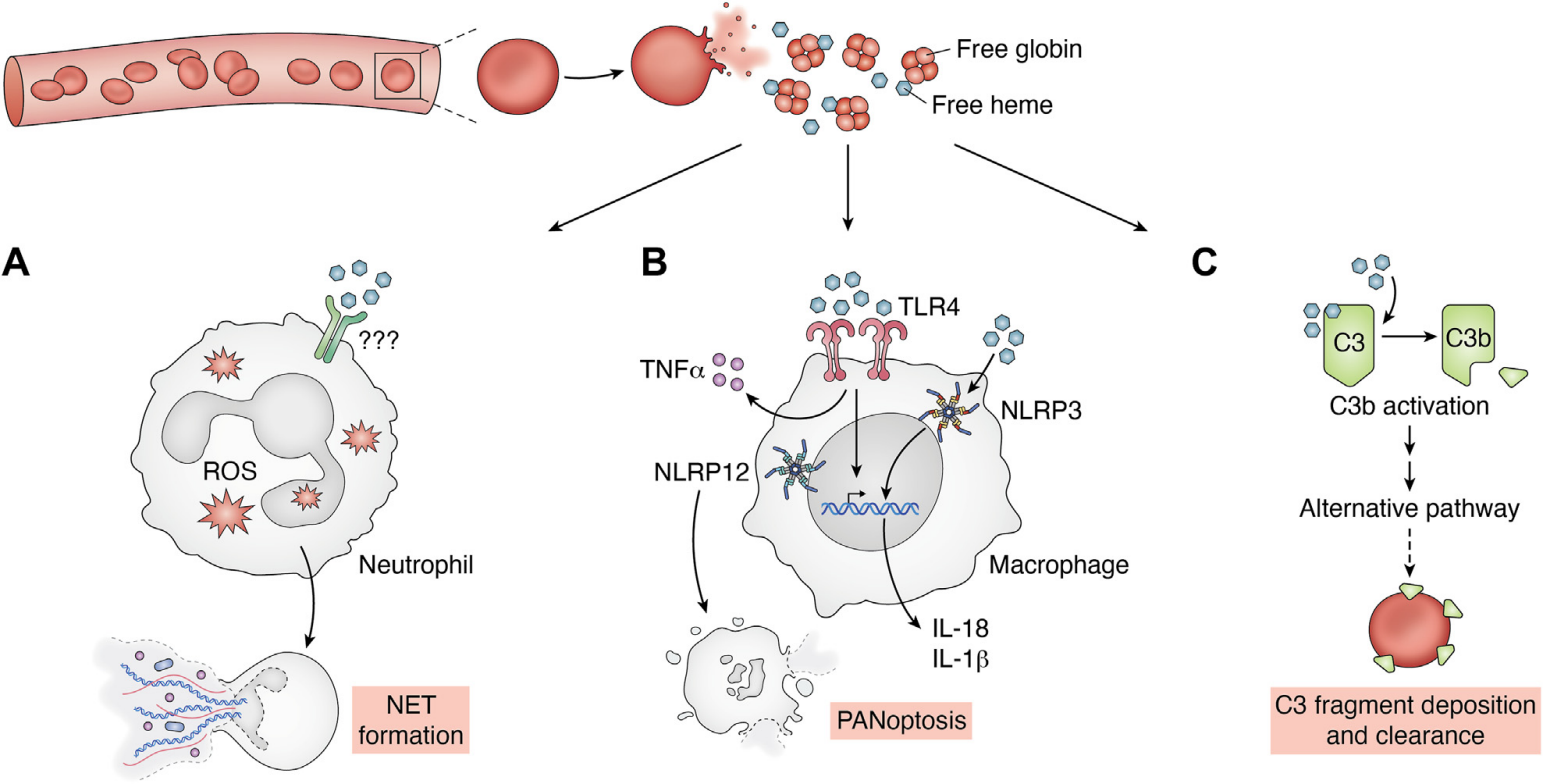
**Regulation of innate immune response by free heme**. *A*, free heme can activate neutrophils to form neutrophil extracellular traps (NETs), which contributes to the pathogenesis of diseases associated with hemolysis, such as sickle cell anemia and malaria. *B*, in conditions with excessive hemolysis, the plasma levels of free hemoglobin (Hb) and heme increases. Free heme binds TLR4 on macrophages to activate them and secrete TNF-α (tissue necrosis factor-α). Heme can also activate inflammasomes (NLRP3 and NLRP12) in macrophages, which results in the secretion of interleukin 1β (IL-1β) and PANoptosis in bone marrow–derived macrophages. *C*, heme interacts with the complement protein C3 and activates it to form C3b, which in turn activates the cascade of the complement system's alternative pathway. The complement fragments can get deposited on erythrocytes and lead to their clearance. TLR4, toll-like receptor 4.

**Figure 5:**
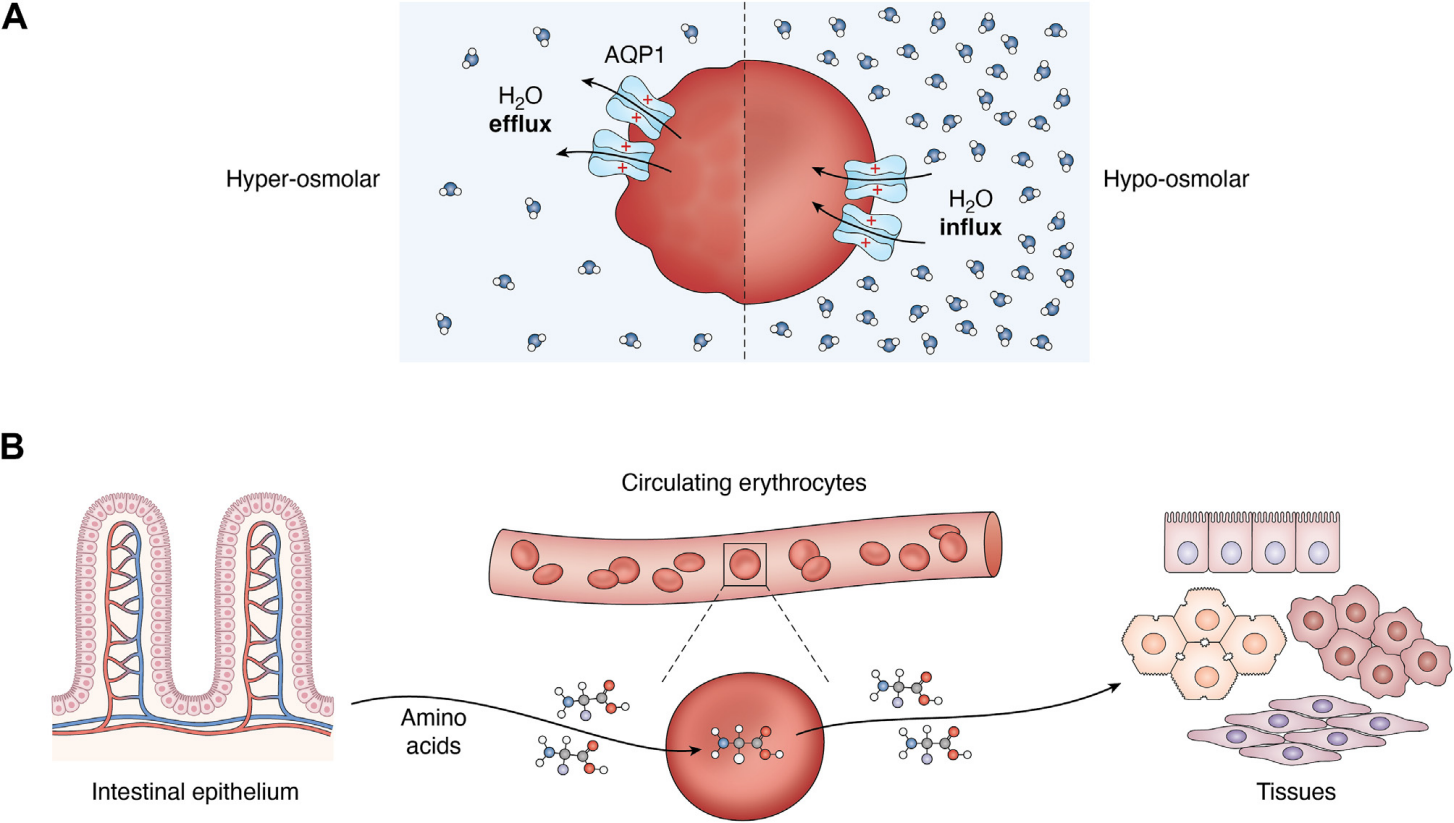
**Role of erythrocytes in water homeostasis and amino acid transport.***A*, erythrocytes contain aquaporin 1 (AQP1) channels on their cell membrane, which help in rapid water flux. In response to a hyperosmolar condition (high osmolarity, less water), erythrocytes shrink to release water to the surroundings and maintain osmolarity (*left panel*). When subjected to a hypo-osmolar environment (less osmolarity, more water), erythrocytes increase their water flux and swell to store the excess water (*right panel*). *B*, erythrocytes express multiple channels, which regulate the transport of amino acids in and out of these cells. Owing to their omnipresent nature, circulating erythrocytes contribute substantially to interorgan amino acid transport by carrying free amino acids from the digestive system and distributing them to different tissues.

The presence of translation and its regulatory molecules, such as miRNAs in erythrocytes, enables these anucleate cells to exhibit multiple functions. The role of erythrocytes in carrying the oxygen-loaded Hb has been a common knowledge for over a century. However, in the past few decades, several erythrocyte functions beyond oxygen transportation have been demonstrated—regulation of local blood flow, regulation of innate immune response and inflammation, water homeostasis, and amino acid transportation. These functions are described later and summarized in Figure 2, Figure 3, Figure 4, Figure 5.

### Erythrocytes regulate local microvascular blood flow

The primary function of erythrocytes is to transport oxygen from the lungs to most of the tissues (except cornea) and to deliver it based on the demand, that is, the level of hypoxia. The delivery of oxygen to tissues is regulated at two levels—(i) release of oxygen from the Hb to tissues based on the pH, temperature, P_CO2_, and the concentration of 2,3 diphosphoglycerate (63) and (ii) the volume of the blood flow to the tissues. The role of erythrocytes in the local regulation of blood flow was identified in the 1990s (64). Nitric oxide (NO), which is a potent vasodilator produced in endothelial cells, binds to the highly conserved residue βCys93 in the β chain of Hb, forming S-nitrosohemoglobin (SNO-Hb). While this binding is favored in oxygenated Hb, deoxygenation induces the release of S-nitrosothiol (SNO) that triggers local vasodilation (65). This phenomenon provides a mechanism for inducing vasodilation and, therefore, oxygen delivery, depending on the oxygen demand of the tissue.

It was believed that the NO of the SNO-Hb was derived from the endothelial cells, and the erythrocytes merely functioned as a passive NO sink to capture and store the NO to be released in tissues with low Po_2_. However, the expression of endothelial NO synthase (eNOS) was demonstrated in erythrocytes by multiple methods, which has been termed red blood cell–eNOS (66, 67, 68, 69). This enzyme, responsible for the production of NO, localizes in the plasma membrane and the cytoplasm of human erythrocytes (70). There is evidence to suggest that mechanical perturbations trigger the activity of erythrocyte eNOS and the production of NO (71). In another study, mice lacking eNOS only in erythroid cells showed enhanced infarct size and left ventricle dysfunction after acute myocardial infarction (72). This study showed the importance of NO derived from erythrocytes in the cardiac tissue response to ischemia.

The physiological significance of SNO-Hb-mediated local vasodilation has been demonstrated using genetically modified mice. Mice with a βCys93Ala (the site of Hb nitrosylation) mutation showed impaired peripheral blood flow autoregulation and skeletal muscle oxygenation. Importantly, these mice not only showed reduced myocardial contractility and increased hypoxia-induced mortality, but they also exhibited signs of myocardial ischemia in normoxic conditions (73). These observations suggest a previously unappreciated role of erythrocytes in the heart function under physiological as well as pathological conditions (74).

Another molecule that executes erythrocyte-mediated vasodilation is ATP. Shear-induced mechanical stretch triggers the release of ATP from erythrocytes (75, 76). This signaling is mediated by Piezo1, a mechanically activated cation channel, which regulates shear-induced Ca^++^ influx (77). The released ATP binds the purinergic G protein-coupled receptors (P2Y) on endothelial cells and induces the synthesis and release of NO, which will, in turn, cause vasodilation (78) (Fig. 2).

### Erythrocytes regulate innate immune response

Owing to their continuous presence in circulation for ∼ 115 days, erythrocytes come in close contact with immune cells and molecules that trigger or regulate immune responses. Several studies have demonstrated the role of erythrocytes in regulating immune response by quenching cytokines as well as cell-free nucleic acids (79, 80).

#### Erythrocytes express toll-like receptors

Elevated levels of cell-free mitochondrial DNA (cf-mtDNA) are observed in blood during pathological conditions, such as sepsis, cancer, trauma, autoimmune conditions, and post-traumatic stress disorder (81, 82, 83, 84). Because of the CpG motif, also found in most bacteria, cf-mtDNA can activate the innate immune system *via* toll-like receptor 9 (TLR9) (85, 86). In a remarkable development in this field, a couple of studies have shown that erythrocytes express TLR9 (79, 87) on their surface. TLR9, which typically recognizes CpG DNA derived from pathogens (88, 89), is usually localized intracellularly in the endocytic vesicles of immune cells (90). However, in erythrocytes, TLR9 is expressed on their surface (79). *Via* TLR9, erythrocytes can bind proinflammatory cf-mtDNA as well as CpG DNA derived from bacterial and *Plasmodium* pathogens. Elevated levels of erythrocyte-bound DNA were observed in sepsis and pneumonia. Because of the absence of downstream signaling pathways, this binding serves as a scavenger under normal conditions (87). Using a rodent lung injury model, it has been demonstrated that the TLR9-mediated quenching of CpG DNA by erythrocytes reduces inflammation and lung injury (79). However, during infections and sepsis, the plasma CpG DNA levels are elevated beyond normal levels. In such conditions, CpG DNA–bound erythrocytes show enhanced erythrophagocytosis because of altered structure and loss of surface CD47, “a marker of self,” leading to anemia. When transfused in naïve mice, CpG DNA–bound erythrocytes triggered the innate immune response. These effects were not observed in erythrocytes lacking TLR9. Thus, erythrocytes work as sentinels in the blood that regulate the activity of the innate immune system (87) (Fig. 3*A*).

Similar to cell-free DNA, cell-free RNA is also found in human blood (91, 92). TLR7 expressed in the vesicles of immune cells serves as a sensor of single-stranded RNA derived from pathogens (93, 94). Interestingly, a recent study has demonstrated the expression of TLR7 on the surface of human erythrocytes (95). Erythrocytes bind exogenous synthetic single-stranded RNAs as well as viral RNAs *via* TLR7. The binding was abrogated when the cells were treated with TLR7 inhibitor enpatoran (95). However, the significance of the binding of RNAs on erythrocytes is not known (Fig. 3*B*).

#### Erythrocytes regulate chemokine response

Much before the discovery of TLR functions in erythrocytes, the role of erythrocytes in the regulation of chemokine functions was established (96, 97). The first report came in 1991 by Darbonne *et al.* (80), who showed that human erythrocytes bind interleukin 8 (IL-8 or CXCL8), which is a potent neutrophil chemotactic factor. This binding was reversible (kilodalton, 5 nM) and did not cause receptor internalization. Also, the bound IL-8 was incapable of causing chemotaxis of neutrophils (80). Two years later, the receptor for IL-8 on erythrocytes was found to be the Duffy blood group antigen (duffy antigen receptor for chemokines [DARCs]), which is a minor blood group antigen as well as the receptor for *Plasmodium vivax* and *Plasmodium knowlesi* (98, 99, 100). Recombinant IL-8 was able to prevent the *Plasmodium* binding and, therefore, entry to human erythrocytes, possibly because of the competition for the same receptor (98, 100, 101, 102). Interestingly, the DARC is quite promiscuous. It can bind several other chemokines, including CXCL1 (melanoma growth stimulatory activity), CCL2 (monocyte chemotactic protein 1), and CCL5 (regulated on activation, normal T expressed, and secreted) (103, 104).

In humans, DARC is encoded by the gene *ACKR1* (atypical chemokine receptor 1). Polymorphisms in *ACKR1* form the basis for various Duffy blood groups (105, 106). Unlike a typical G protein-coupled receptor, DARC lacks the consensus motif DRYLAIV in the second intracellular loop required for signaling *via* G proteins. Therefore, the binding of chemokines to DARC on the surface of erythrocytes does not trigger any signaling inside the cells (107). Thus, it serves as a “sink” for chemokines that controls the levels of soluble chemokines in plasma. This hypothesis was tested using blood from individuals with a polymorphism in the GATA-1 promoter region (T-46C substitution) of the *ACKR1*. This mutation completely abolishes the expression of DARC in erythroid cells in homozygous individuals and partially in heterozygous individuals (108, 109). Plasma levels of CXCL8 and CCL2 in humans homozygous for this polymorphism were higher than in heterozygotes following *in vitro* lipopolysaccharide (LPS) stimulation of their whole blood. Similarly, *ACKR1* knockout mice showed higher levels of plasma CCL2 after *in vitro* LPS stimulation of whole blood (110). These observations show that DARC negatively regulates the soluble chemokine concentrations and, therefore, supports the “sink” hypothesis. In addition, erythrocytes delay the clearance of bound chemokines from the blood, serving as their “reservoir.” This may prolong the proinflammatory effects of those chemokines (96). Thus, erythrocytes function as a “rheostat” of the innate immune response by quenching the key triggers (nucleic acids) and mediators (chemokines) (Fig. 3*C*).

#### Regulation of innate immune response by Hb-derived free heme

Hb, the major constituent of erythrocytes, is the oxygen-carrying pigment made up of two alpha globin chains (encoded by *HBA1* and *HBA2*), two beta globin chains (encoded by *HBB*), and four heme groups. Heme is made up of a porphyrin ring complexed with ferrous iron. Lysis of erythrocytes inside the vascular compartment leads to the release of Hb into the plasma. Extracellular Hb gets quickly oxidized, and this results in the release of heme. Serum proteins such as haptoglobin (111) and hemopexin (112) bind Hb and heme, respectively, aiding their clearance from the circulation. However, in certain pathological conditions, such as sickle cell disease, hemolytic anemia, and sepsis, the plasma levels of Hb and heme increase beyond the ability of haptoglobin and hemopexin to neutralize them. Unfortunately, heme happens to be a potent proinflammatory molecule when it is present in the extracellular compartment. It can trigger chemotaxis and activation of neutrophils leading to neutrophil extracellular traps (Fig. 4*A*). This property of heme contributes to the pathogenesis of diseases associated with hemolysis, such as sickle cell disease and malaria (113, 114, 115, 116). The mechanisms of proinflammatory effects of Hb-derived extracellular heme are described later.

##### Heme and TLR4

Free heme can activate macrophages to secrete tumor necrosis factor-alpha. This effect was not observed in macrophages derived from mice lacking CD14 (a coreceptor of bacterial LPS that activates innate immune system) or MyD88 (an adapter protein that is important for downstream signaling in TLR pathway) or from a mouse (C3H/HeJ) expressing mutant TLR4 (117) (Fig. 4*B*). Endothelial cells are also targets of heme. In sickle cell mice, the extracellular heme released after hemolysis activates endothelial cells *via* the TLR4 signaling pathway. This leads to leukocyte adhesion and vaso-occlusion, which is a hallmark of human sickle cell disease (118). These results suggest that heme is an endogenous ligand of TLR4. However, the direct interaction between TLR4 and heme has not been demonstrated so far.

##### Heme and inflammasomes

Free heme has also been shown to activate inflammasomes in macrophages and endothelial cells (119, 120, 121). When treated with heme, LPS-primed murine bone marrow–derived macrophages showed maturation and secretion of interleukin-1β (IL-1β), which is an indication of inflammasome activation. This was also observed when heme was injected into the peritoneal cavity of mice. The heme-induced maturation of IL-1β was not observed in macrophages derived from mice devoid of inflammasome components, that is, NLRP3 (nucleotide-binding domain and leucine-rich repeat-containing family and pyrin domain containing 3), Asc (Apoptosis-associated speck-like protein containing a caspase recruitment domain), and Caspase-1, demonstrating that heme indeed activates inflammasomes. The iron of the heme was essential for all these effects (119). Ferryl-Hb, an oxidized form of Hb, has also been shown to activate inflammasomes in LPS-primed cells leading to maturation and secretion of IL-1β (120). In a recent study, heme, along with LPS, has been found to cause cell death *via* NLRP12-PANoptosome in bone marrow–derived macrophages (122) (Fig. 4*B*).

##### Heme and complement system

The complement system is a part of the innate immune system, which enhances its effectiveness. This system consists of several serum proteins normally present in circulation in an inactive state. Heme interacts with the complement protein C3 and activates it to form C3b, which in turn triggers the cascade of activation of the complement system's alternative pathway (123). When oxidized, hematin, another iron-containing pigment derived from Hb, can also activate the alternative pathway of the complement system. This pigment causes C3 activation and deposition of its fragments on erythrocytes. These fragments act as an opsonin (molecules that coat cells to enhance phagocytosis-mediated destruction) and may lead to the clearance of erythrocytes contributing to anemia associated with diseases, such as malaria, sickle cell disease, and β-thalassemia (124, 125) (Fig. 4*C*).

All these findings strongly suggest that heme serves as a death-associated molecular pattern that can trigger the activation of the innate immune system and inflammatory response, which can be beneficial if it is controlled or harmful if it is unchecked (126, 127).

### Water homeostasis by erythrocytes

Erythrocytes can rapidly swell or shrink according to the osmolality of their environment. This is possible because of the rapid flux of water into and out of erythrocytes. The water channel of erythrocytes that is primarily responsible for this rapid water flux is aquaporin-1 (AQP1) (128, 129). Erythrocytes exposed to the natural hyperosmolar environment in the medulla of kidneys are shrunken compared with those in the cortex. This shrinkage was not observed in *AQP1* knockout mice (130). Each erythrocyte has about 2 × 10^5^ copies of AQP1 per cell (131). Because of these channels, erythrocytes can rapidly swell to store excess water from a hypo-osmolar environment or shrink to release water into a hyperosmolar environment. Thus, it can regulate the water homeostasis and osmolality of the blood (130) (Fig. 5*A*).

### Amino acid transport by erythrocytes

As mentioned previously, all standard protein-coding amino acids can enter mammalian erythrocytes *via* transporters or unknown mechanisms (36, 37, 38, 39, 40, 41, 42, 43, 44, 45, 46, 47, 48, 49, 50). Erythrocytes appear to use multiple mechanisms for amino acid uptake. Kinetic studies have revealed that neutral amino acids make use of Na^+^-dependent as well as Na^+^-independent pathways for transport. The transport of bulkier neutral amino acids, such as leucine and isoleucine, relies on the Na^+^-independent pathway, whereas the transport of glycine and alanine is predominantly driven by the Na^+^-dependent pathway (43). In the case of glycine, erythrocytes use the anion exchange transporter (band 3) (36).

Several studies conducted as early as the 1960s provided strong evidence to suggest that mammalian erythrocytes transport amino acids to tissues (132, 133). By measuring levels of multiple amino acids in the plasma and blood cells derived from arteries and veins, Felig *et al.* (134) concluded that blood cells, presumably erythrocytes, contribute substantially to interorgan amino acids transfer from the gut to other tissues. Importantly, the concentration of several amino acids in the cells of venous blood was less than that in the cells of arterial blood, which suggests that blood cells contribute to the amino acids absorbed by tissues (134). This study performed the amino acid measurements in whole blood cells, not in pure erythrocytes. Nevertheless, given their abundance (about 45% of blood volume) and their ability to take in amino acids, erythrocytes are likely to play a key role in the transport of amino acids from the digestive system to other tissues *via* liver (135, 136) (Fig. 5*B*).

## Potential clinical applications

**Figure 6:**
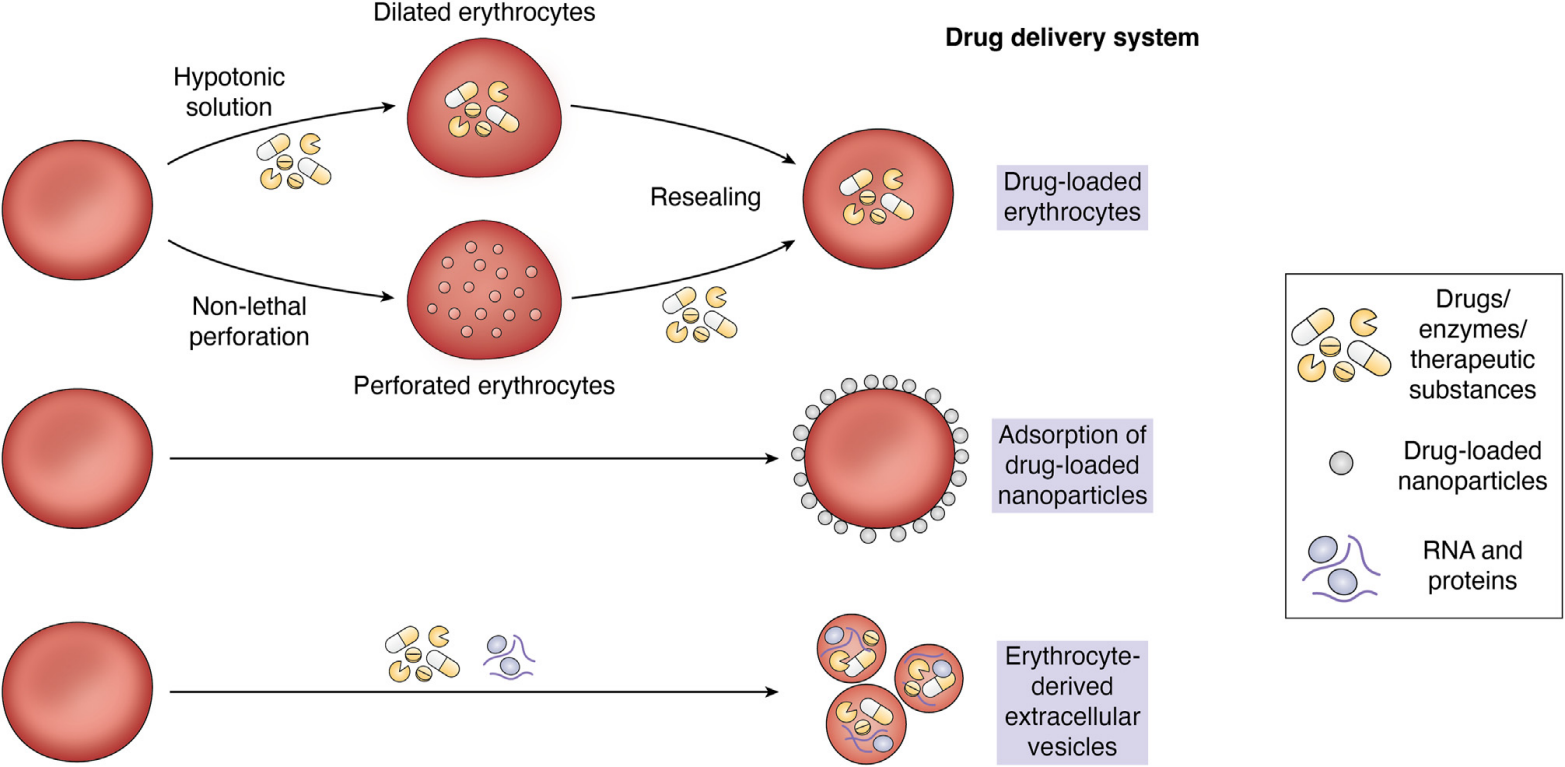
**Erythrocytes as drug delivery vehicles**. Multiple methods have been developed to use erythrocytes as a drug delivery system. Erythrocytes can be loaded with drugs by hypotonic lysis and nonlethal perforation on their membrane by chemicals such as amphotericin B. Erythrocyte-derived extracellular vesicles (EVs) can also be used as vehicles to carry therapeutic molecules. Erythrocyte-hitchhiking nanocarriers can also be used for the effective delivery of therapeutic molecules.

Erythrocytes can be easily accessed. The number, composition, and shape of erythrocytes are altered during multiple diseases. The diagnostic value of these parameters of erythrocytes in the detection of certain infectious diseases (*e.g.*, malaria), anemia, chronic kidney disease, and diabetes is well established. Recent developments have demonstrated the diagnostic value beyond these diseases based on the molecular composition of erythrocytes. Furthermore, erythrocytes are being explored as vehicles for the delivery of therapeutic molecules (Fig. 6).

### Erythrocyte miRNAs and proteins as diagnostic markers

The profile of miRNAs in human erythrocytes changes in several clinically relevant conditions. For example, high levels of miR-144 have been observed in erythrocytes of a subset of sickle cell disease patients with severe anemia (57). The levels of miRNAs, miR-96, miR-150, miR-196a, and miR-197, in erythrocytes decrease in stored blood (137). Members of the let-7 family miRNAs are downregulated in erythrocytes infected with *Plasmodium* (138). Changes in erythrocyte miRNA profile have been observed in other pathological conditions, such as lung carcinoma (139), immunoglobulin A neuropathy (140, 141), and relapsing-remitting multiple sclerosis (142). These observations show that the erythrocyte miRNA expression profile has the potential to be used as a diagnostic marker or a prognostic marker.

Alzheimer's disease is a progressive neurodegenerative condition characterized by misfolding and pathological aggregation of β-amyloid and tau proteins (143). These proteins can cross the blood–brain barrier, and the aggregates can adhere to the surface of the erythrocytes. These aggregates on erythrocytes can be detected by atomic force microscopy. The morphology of the aggregates is dependent on the age of the patient and the stage of Alzheimer's disease and, therefore, can be used as a diagnostic marker (144, 145). Erythrocytes are the major source of alpha-synuclein outside the central nervous system. The levels of alpha-synuclein in the membrane fractions of erythrocytes can serve as a biomarker for Parkinson's disease (146, 147).

### Therapeutic targeting of erythrocyte RNAs

The presence of RNAs and translation in erythrocytes provides new therapeutic avenues. Their abundance and easy accessibility make erythrocytes easy targets for the delivery of therapeutic molecules. For example, molecules such as ataluren or specific ASOs that can induce translational readthrough across disease-causing premature stop codons can be used in the treatment of thalassemia, hereditary spherocytosis, and other genetic diseases that affect erythrocytes caused by nonsense mutations (148, 149, 150). Mimics of miR-451 and miR-let-7i can potentially be used in the treatment of malaria. Also, mRNAs of therapeutic value (*e.g.*, insulin-coding mRNA) can potentially be delivered to erythrocytes as they can be translated into proteins during the entire lifespan of erythrocytes. This strategy would require less frequent deliveries compared with the conventional delivery strategies *via* intravenous, intramuscular, or subcutaneous routes (*e.g.*, insulin).

### Erythrocytes as drug delivery vehicles

Erythrocytes reach most of the human tissues. Because they lack all membrane-bound organelles, erythrocytes have plenty of space for exogenous molecules compared with other cells. They can protect the exogenous molecules from the components of blood (*e.g.*, nucleases) and detection by the immune system. These properties, along with their abundance and ∼115 days of lifespan, make erythrocytes an ideal system for drug delivery (Fig. 6). Furthermore, with erythrocytes, there is no biocompatibility or toxicity issue to deal with.

Successful attempts were made in the early 1970s to load erythrocytes with enzymes, such as uricase, beta-glucosidase, and beta-galactosidase. Hypotonic lysis of erythrocytes, which results in ghost cells, was employed for the loading of these enzymes. Such enzyme-loaded erythrocyte ghosts will be phagocytosed, making this method a promising delivery system to target phagocytic cells of reticuloendothelial system (151, 152). Even live erythrocytes can be used as carriers of drugs. Amphotericin B has been used to make nonlethal perforations on the erythrocyte membrane. By this method, the anticancer drug daunomycin was loaded in human and mouse erythrocytes without hemolysis (153). The *in vivo* feasibility and efficacy of these methods are yet to be established. A multicenter, randomized, double-blind, placebo-controlled phase 3 trial of intraerythrocyte delivery of dexamethasone for the treatment of ataxia telangiectasia (a neurodegenerative genetic disease caused by mutations in *ATM* gene and characterized by poor motor coordination and dilation of small blood vessels) patients was conducted recently (154). Dexamethasone induces noncanonical splicing in *ATM* leading to skipping of the mutations and generation of truncated, yet functional protein (155). Although the primary efficacy endpoint was not met after the 6-month treatment in the entire study population, there appeared to be a potential benefit of treatment in a subgroup of patients aged 6 to 9 years. The trial is being continued for patients of this age group.

### Erythrocyte-derived extracellular vesicles as drug delivery vehicles

Erythrocyte-derived extracellular vesicles (EVs) are membrane-bound vesicles secreted by erythrocytes into extracellular space during normal aging. They carry several cellular components and serve as a means of communication between cells (for review on EVs, read Refs. (156, 157). EVs derived from erythrocytes have been used as vehicles to carry potential therapeutic molecules both *in vitro* and *in vivo*. Usman *et al.* (156) used erythrocyte-derived EVs to deliver miR-125b-targeting ASOs into human leukemia cells, which resulted in reduced proliferation. The same EVs were able to reduce the progression of breast cancer and leukemia in corresponding xenograft nude mice models. In the same study, authors used erythrocyte-derived EVs to deliver Cas9 mRNA and guide-RNA into leukemia cells to achieve target-specific genome editing (156). In another study, erythrocyte-derived EVs were used to deliver siRNAs targeting *MSTN* mRNA (encodes myostatin). When injected intramuscularly, these EVs reduced the expression of myostatin, prevented cachexia, and improved muscle growth in cancer-bearing mice (157). Agonists of the cytoplasmic RNA sensor retinoic acid–inducible gene-I delivered through erythrocyte-derived EVs were able to activate the retinoic acid–inducible gene-I pathway and reduce breast cancer growth in nude mice (158). Pham *et al.* (159) have achieved targeted drug delivery using erythrocyte-derived EVs. Using protein ligases sortase A and OaAEP1 ligase, the authors of this study conjugated erythrocyte-derived EVs with a peptide or a nanobody, which binds the epidermal growth factor receptor. These conjugated EVs accumulated in epidermal growth factor receptor–positive cells. Delivery of paclitaxel by these conjugated EVs enhanced anticancer treatment efficacy in xenograft tumors in immunodeficient NOD scid gamma mice (159) (Fig. 6).

### Drug delivery by erythrocyte hitchhiking

One of the major problems in nanocarrier-mediated drug delivery is the predominant uptake by the liver and spleen, which results in insufficient delivery to target organs. To circumvent this issue, Anselmo *et al.* (160) noncovalently adsorbed nanocarriers onto erythrocytes and injected these erythrocyte-hitchhiking nanocarriers in mice to achieve ∼7-fold higher accumulation in lungs compared with just nanocarriers. This method prolongs the circulation time and increases the delivery of the nanocarriers to organs/tissues downstream of the injection site. This has been demonstrated in mice, pigs, and *ex vivo* in human lungs without damaging erythrocytes or causing toxicity (161, 162, 163). Erythrocyte hitchhiking method has been used for the efficient delivery of adeno-associated virus to the lungs. Interestingly, this method reduced the neutralization of adeno-associated viruses by neutralizing antibodies during subsequent administration (164) (Fig. 6).

A similar technology termed erythrocyte leveraged chemotherapy was used to deliver chemotherapeutic drug doxorubicin enclosed in nanocarriers. This method extended the circulation time of the drug nanocarriers, and the drug delivery was ∼10-fold higher compared with free nanocarriers. In mice (B16F10-Luc metastasis model), the erythrocyte leveraged chemotherapy platform achieved a substantial reduction in lung metastasis and improved survival compared with free nanocarrier treatment (165). Ukidve *et al.* (166) used a similar method termed erythrocyte-driven immune targeting to adsorb antigenic nanocarriers onto erythrocytes. This led to improved antibody response and higher memory T-cell response against that antigen, suggesting that erythrocyte hitchhiking can be an effective method of immunization (166).

## Conclusion

Erythrocytes reach almost all tissues of our body. Their oxygen transport function is closely coupled with the cardiovascular system. Hence, it is not surprising that they have evolved the ability to regulate the regional blood flow as per the oxygen needs of the tissues. Also, because of their close association with immune cells in circulation, it is only natural that erythrocytes can influence immune responses. Furthermore, evidence to support their role in water homeostasis and amino acid transport are mounting. To perform vital oxygen transport and other functions for a period of ∼115 days demands a fresh supply of proteins. Recent discoveries of RNAs, protein synthesis, and degradation show that erythrocytes are capable of generating their own proteins, though at a low level. With these developments in the field, the long-held notion that erythrocytes are mere transporters of respiratory gases no longer holds true. Yet, several intriguing questions remain to be addressed. It is not known how RNAs inside erythrocytes stay for the entire duration of the erythrocytes. They express several membrane receptors. However, it is not clear if there are any downstream signaling pathways. If yes, what do they activate finally in the absence of the nucleus? Translatome analyses indicate the expression of transcription factors like *c-Jun*, *Fos*, and so on in erythrocytes (51). What role they serve in these anucleate cells is a mystery. Can erythrocytes be used to express exogenous mRNAs for therapeutic purposes? Do they uptake RNAs and proteins generated by other cells for their own use? We can anticipate exciting developments in this field in the near future.

## Conflict of interest

The authors declare that they have no conflicts of interest with the contents of this article.
